# A Trends Analysis and Scoping Review: Minimal Invasive Extracorporeal Circulation

**DOI:** 10.1007/s13239-026-00845-x

**Published:** 2026-06-25

**Authors:** Doğuş Hemşinli, Sedat Ozan Karakişi, Hülya Kılıç

**Affiliations:** 1 Faculty of Medicine, Department of Cardiovascular Surgery, Recep Tayyip Erdoğan University, Rize, Türkiye; 2 Faculty of Medicine, Department of Medical Biochemistry, Recep Tayyip Erdoğan University, Rize, Türkiye

**Keywords:** MiECC, Minimal ınvasive extracorporeal circulation, Minimal extracorporeal circulation, Mini-CPB, Mini cardiopulmonary bypass, Miniaturized extracorporeal circulation, Bibliometrics, Trend analysis, Cardiopulmonary bypass, Extracorporeal circulation

## Abstract

**Purpose:**

The minimal invasive extracorporeal circulation (MiECC) was developed as abiocompatible alternative to conventional cardiopulmonary bypass (cCPB), intending to mitigate haemodilution, lessen the systemic inflammatory response, and enhance organ protection. AQ2 Although the evidence base has grown over the past 30 years, there has been no systematic mappingof the evolution of research activity and its results.

**Methods:**

The objectives are to perform a detailed analysis of the temporal, geographical, methodological, and clinical trends within MiECC research published between 1990 and 2025. Studies involving original human clinical data (randomized controlled trials, cohort studies, observational reports, methodological or protocol papers) that reported perioperative or clinical outcomes of MiECC were included. Exclusion criteria comprised reviews, meta-analyses, editorials, letters, guidelines, animal studies, and conference abstracts lacking full text. A comprehensive search of PubMed/MEDLINE, Web of Science, Scopus, and Cochrane databases was conducted up to September 2025 to gather evidence. Reference lists of included articles and relevant reviews were also screened. Data were extracted on publication year, country, sample size, study design, surgical procedure, and reported outcomes. Two reviewers independently charted the data, and discrepancies were resolved by a third reviewer.

**Results:**

A total of 151 studies were identified, of which 128 met eligibility criteria. Publication activity increased substantially after 2002 and peaked in 2021, with major contributions from European centres (Germany, Italy, Switzerland, Greece, and the Netherlands). Methodological/protocol papers and Randomize Controlled Trials (RCT) predominated (respectively, n = 37, n = 36). Most studies focused on coronary artery bypass grafting (CABG), while valve surgery and paediatric populations were underrepresented. Reported outcomes shifted over time: early studies emphasised transfusion and inflammatory markers, whereas more recent investigations increasingly examined renal function, neurological complications, and survival. Subgroup analyses suggested notable benefits in elderly patients, those with renal dysfunction, and individuals with low ejection fraction (EF).

**Conclusion:**

MiECC research has progressed from feasibility series to large multicentre trials, generating evidence for reduced transfusion requirements, attenuated inflammatory response, and better organ protection, though no clear mortality benefit has been demonstrated.

**Graphical Abstract:**

**Figure:**
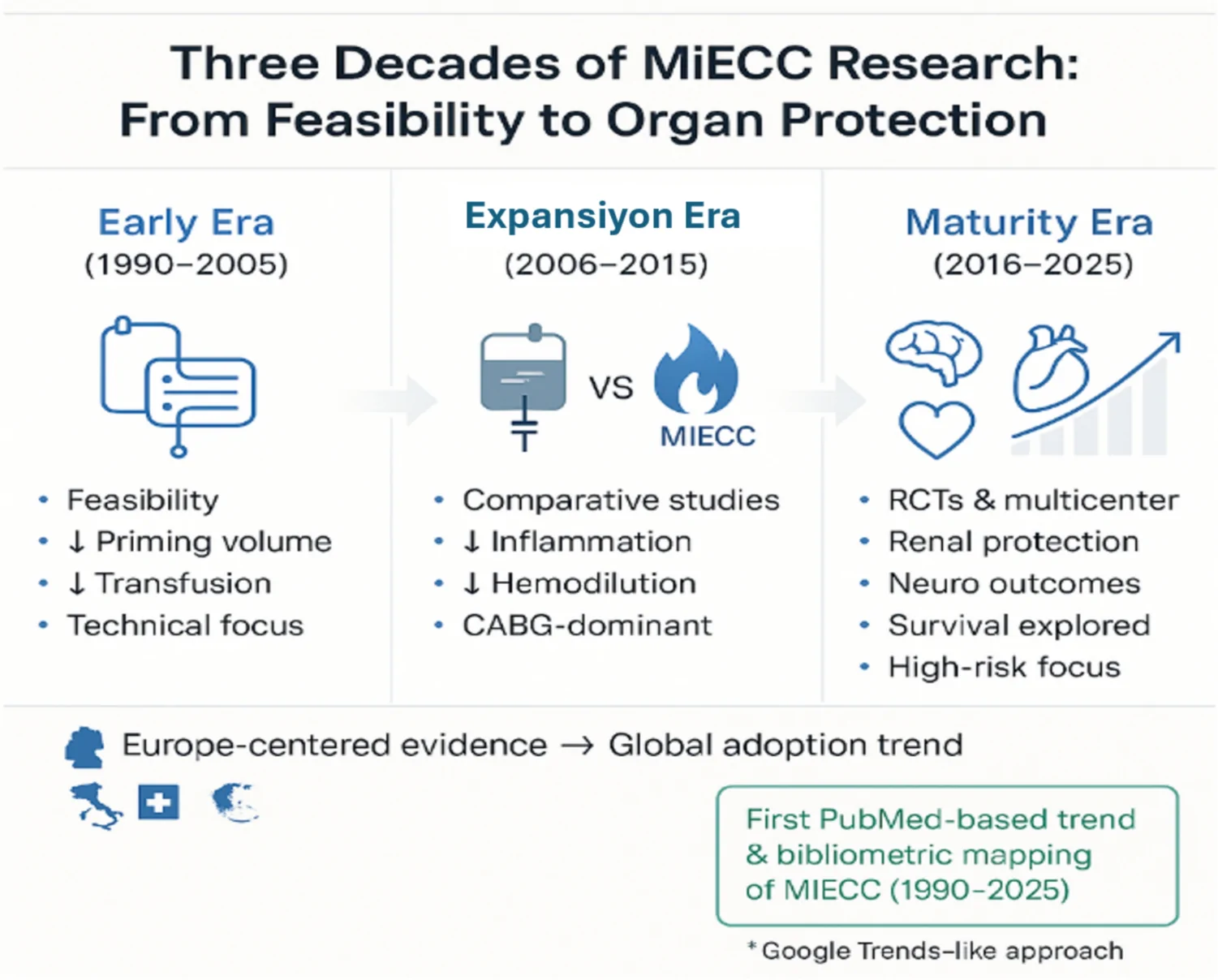

**Supplementary Information:**

The online version contains supplementary material available at https://doi.org/10.1007/s13239-026-00845-x.

## Introductıon

Cardiopulmonary bypass (CPB) has been a cornerstone of modern cardiac surgery ever since its first successful application by Gibbon in 1953. Despite its revolutionary impact, cCPB is associated with recognised complications, including haemodilution, systemic inflammatory response, coagulopathy, organ dysfunction, and an increased need for blood transfusions [1–7]. These limitations have prompted the ongoing search for safer, more biocompatible perfusion strategies [8–10]. Introduced in the 1990 s, MiECC is a refined alternative designed to reduce priming volume, limit blood–air and blood–foreign surface contact, and enhance biocompatibility [11]. Early studies suggested that MiECC reduces the need for transfusions, reduces systemic inflammation and provides better organ protection [12–16]. The principal technical differences between MiECC and cCPB are summarised in Table 1. Unlike cCPB, which operates as an open circuit with a venous reservoir, cardiotomy suction, and significant blood-air contact, MiECC employs a fully closed circuit design with reduced priming volume, eliminated blood-air interface, and enhanced biocompatible surface coating, resulting in attenuated haemodilution and a diminished systemic inflammatory response.

**Table 1:** Key technical differences between MiECC and cCPB

Feature	MiECC	cCPB
Priming volume	< 500 mL	> 1000 mL
Venous reservoir	Absent	Present
Cardiotomy suction	Absent or minimised	Present
Blood-air contact	Eliminated	Present
Circuit surface coating	Fully biocompatible	Partial or absent
Haemodiluton	Minimal	Significant
Systemic inflammatory activation	Attenuated	Pronounced
Circuit type	Closed	Open

Over the past three decades, research has progressed from small-scale feasibility reports to large, multicentre, randomised, controlled trials, and MiECC has been formally standardised through the Minimal Invasive Extracorporeal Circulation Type and Safety Classification System (MiECTiS). Nevertheless, the existing literature on MiECC remains heterogeneous. Studies vary widely in terms of patient populations, surgical procedures, circuit configurations, and reported outcome measures. Furthermore, the focus of research has shifted over time; while initial studies emphasised transfusion and inflammatory markers, more recent studies have increasingly examined renal outcomes, neurological complications, and long-term survival [17–81]. Despite this evolution, there has been no comprehensive evaluation of how MiECC research has developed across different regions, time periods, and methodological designs (911-140).

To the best of our knowledge, this is the first PubMed-based trend and bibliometric analysis to focus specifically on MiECC. By applying a 'Google Trends'-like approach to PubMed data, we introduce a methodological innovation that systematically maps the trajectory of research activity and reported clinical endpoints in MiECC over the last three decades. This study provides a quantitative and qualitative overview of how MiECC has evolved from an early feasibility phase to mature multicentre RCT, and how its clinical impact has been investigated in relation to transfusion needs, inflammatory response, renal function, neurological outcomes, and survival.

## Methods

### Search Strategy

**Fig. 1:**
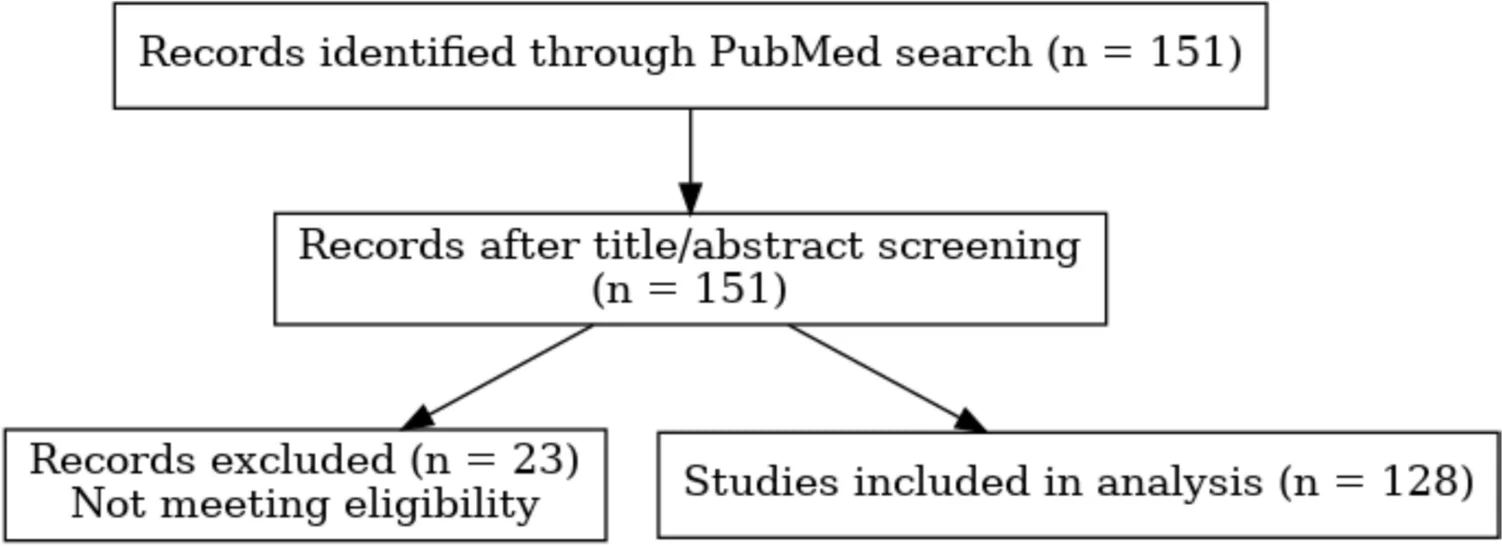
PRISMA-ScR flow diagram

To identify MiECC research, a comprehensive search of the PubMed/MEDLINE, Web of Science, Scopus, and Cochrane databases was performed, up to September 1, 2025. To improve accuracy, we implemented a search algorithm specifically designed to focus on the title field, excluding secondary research and non-original content. The final search code was as follows for Pubmed: *(miecc[ti] OR "Minimal Invasive Extracorporeal Circulation"[ti] OR "minimal extracorporeal circulation"[ti] OR "mini-CPB"[ti] OR "mini cardiopulmonary bypass"[ti] OR "miniaturized extracorporeal circulation"[ti] OR "miniaturised extracorporeal circulation"[ti]) AND humans[MeSH Terms] NOT (Review[Publication Type] OR "Systematic Review"[Publication Type] OR Meta-Analysis[Publication Type] OR Editorial[Publication Type] OR Letter[Publication Type] OR Comment[Publication Type] OR Guideline[Publication Type]).* This strategy was created to include only original human studies with primary data and to exclude reviews, meta-analyses, editorials, letters, comments, and guidelines. Furthermore, the reference sections of the included articles and pertinent reviews were manually examined to identify any supplementary eligible studies. The review process was conducted in accordance with the PRISMA-ScR guidelines. A PRISMA-ScR flow diagram (Figure 1) summarizes the study selection process, and the completed PRISMA-ScR checklist is provided as Supplementary Material (Suppl1_PRISMA-ScR-Fillable-Checklist_10Sept2019.docx).

### Eligibility Criteria

The study encompassed original clinical research evaluating the use of MiECC. Eligible studies included those conducted in adult or paediatric patient groups undergoing cardiac surgery with CPB, where MiECC (types I–IV) or similarly minimised extracorporeal circulation circuits were used and compared with cCPB or other alternative methods. RCT, prospective or retrospective cohort studies, and case series were considered. In addition, two in vitro studies published in 2025 were included, as they addressed the topic from a different perspective. Studies were required to report at least one clinical, biochemical, or technical outcome, such as perioperative bleeding and transfusion requirement, inflammatory and coagulation markers, renal and neurological complications, myocardial protection parameters, or survival. Only articles published in English between 1990 and 2025 were included. Exclusion criteria comprised animal experiments, narrative reviews, systematic reviews, meta-analyses, guidelines, editorials, commentaries, and conference abstracts without full text. In total, 151 studies met the initial search criteria, but 23 were excluded for not fulfilling the predefined eligibility criteria.

### Data Extraction

From each eligible study, data were extracted using a standardised data extraction form. The dataset was created in Excel format. Two researchers (DH, HK) independently reviewed the data, and any discrepancies were resolved by a third researcher (SOK). Extracted variables included chronological and geographical distribution of the studies, sample size of the analysed population, surgical procedure, and comparator groups (cCPB, off-pump surgery, or different MiECC configurations). Primary and secondary outcomes comprised perioperative bleeding, transfusion requirements, inflammatory and coagulation markers, renal and neurological complications, myocardial protection parameters, survival, and length of Intensive Care Unit or hospital stay. In addition, technological components (e.g., MiECC type, circuit coating, pump and oxygenator used) were recorded and incorporated into the trend analysis to capture temporal changes. The resulting dataset has been provided as a supplementary Excel file (Suppl2_MiECC_Data_Extraction_Template). The overall advantage classification presented in Table 2 (Strong, Moderate, or Limited evidence) was determined based on the consistency, volume, and methodological quality of the supporting evidence identified during data extraction, rather than a formal meta-analytic synthesis, in keeping with the descriptive nature of this scoping review.

**Table 2:** Subgroup outcomes comparing MiECC with cCPB

Subgroup	Key Findings (MiECC vs cCPB)	Overall Advantage*	Key References
CABG	↓ Transfusion, ↓ Hemodilution, ↓ Inflammatory response, Safe in high-risk patients	Strong	16, 29, 36, 60, 70, 73
Valve surgery	↓ Hemodilution, ↓ Biomarkers; technical differences may affect outcomes	Moderate	19, 35, 42, 65, 102
Elderly patients	↓ Transfusion, ↓ Inflammatory response, ↓ Perioperative complications	Strong	98, 106
Renal dysfunction	↓ Creatinine increase, Renal protection	Strong	46, 51, 74, 136
Low EF (<35%)	Hemodynamically tolerated, ↓ Troponin (better myocardial protection)	Moderate	15, 120
Pediatric population	↓ Hemodilution, ↓ Transfusion need; limited sample size	Limited evidence	14, 82, 133

^*^Strong: consistent findings across ≥ 3 RCT or large prospective studies with concordant results. Moderate: supportive findings from ≥ 2 studies with heterogeneous populations, limited sample sizes, or technical confounders. Limited evidence: findings from ≤ 1 small study or case series with insufficient power for definitive conclusions. RCT: randomised controlled trial; MiECC: minimally invasive extracorporeal circulation; cCPB: conventional cardiopulmonary bypass; CABG: coronary artery bypass grafting; EF: ejection fraction.

## Results

### Origins of MiECC

The idea of minimising extracorporeal circulation dates back to the early 1990 s. One of the first reports on this subject was published in 1990 by Honĕk and colleagues from Czechoslovakia. The authors described the use of a miniaturised extracorporeal circulation system in infants [10]. By utilising the smallest commercially available oxygenator and positioning the circuit close to the operating table, they succeeded in reducing tubing length and priming volume. In their series of ten paediatric cases, the minimised circuit achieved a mean priming volume of 420 mL compared with 671 mL in the control group. The control group required a substantially larger volume of transfused blood (~500 mL/patient) to achieve a comparable packed cell volume. Based on the authors' own conclusion, this circuit configuration made perfusion without donor blood possible in the majority of children and infants with an elevated packed cell volume, consistent with contemporary trends at the time. As only the abstract of this study is available, these findings should be interpreted with appropriate caution. Although no significant differences were observed in postoperative outcomes, this pioneering study demonstrated the feasibility of reducing priming volume and avoiding allogeneic transfusion. In this respect, it is regarded as one of the earliest clinical steps—consistent with current trends—that later evolved into the concept of MiECC[11].

### Number of Studies by Year and Country

**Fig. 2:**
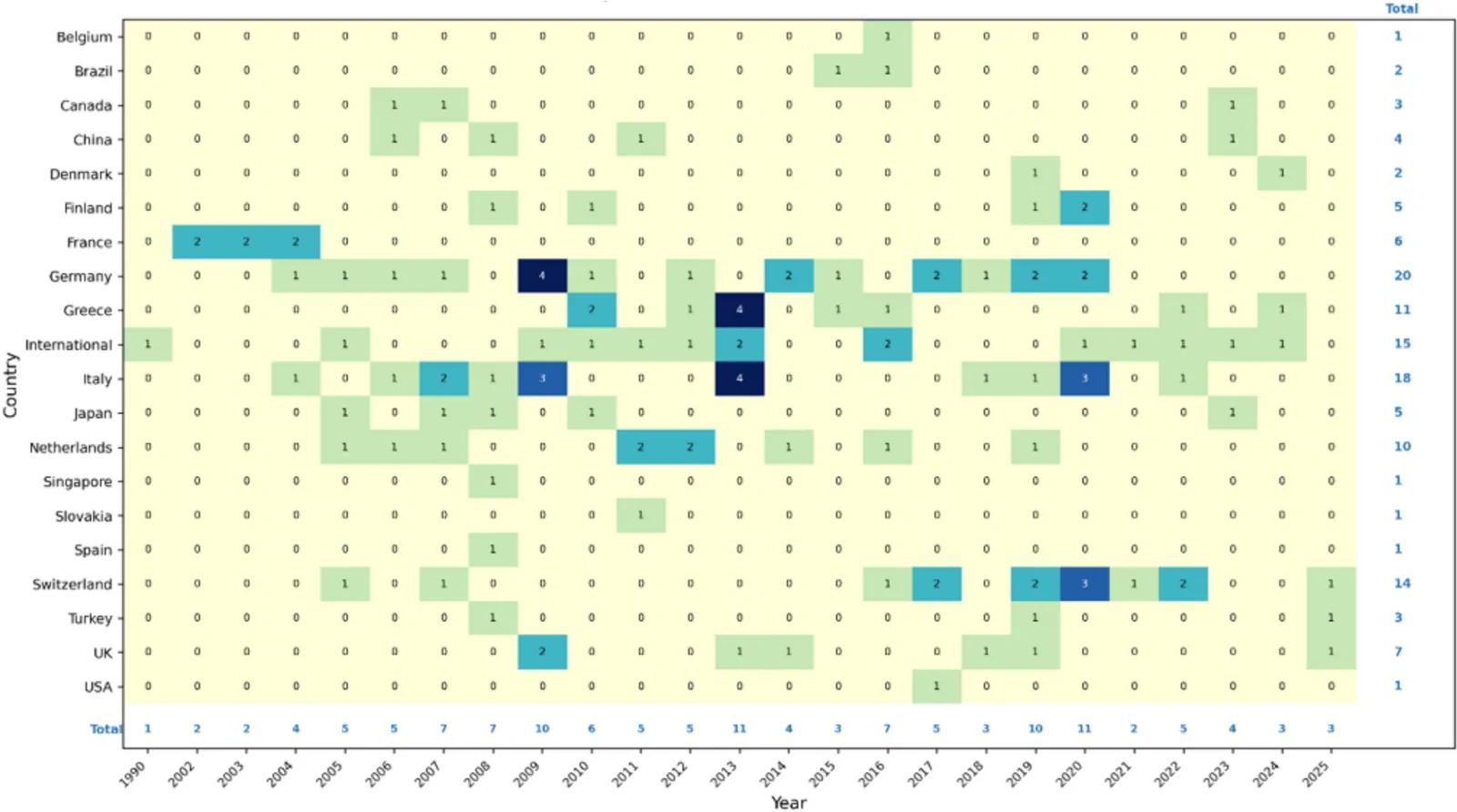
The distribution of publication years and counts by country was visualised using a heatmap to illustrate geographical and temporal patterns in MiECC research. All contributing countries are listed individually regardless of publication volume, as each national contribution is considered meaningful within the context of a bibliometric mapping analysis. The "International" category denotes multi-country collaborative studies that could not be attributed to a single nation. For geographical reference: European countries include Belgium, Denmark, Finland, France, Germany, Greece, Italy, Netherlands, Slovakia, Spain, Switzerland, Turkey, and United Kingdom; Asian countries include China, Japan, and Singapore; North American countries include Canada and USA; South American countries include Brazil

An examination of the temporal distribution of literature concerning MiECC indicates that the initial study originated in the Czechoslovakia in 1990[10]. It should be noted that this publication is attributed to Czechoslovakia, reflecting the political entity in existence at the time of publication. Following the dissolution of Czechoslovakia in 1993, subsequent contributions from authors at institutions in the successor states are attributed to the Czech Republic or Slovakia according to their stated affiliations. However, subsequent to this initial endeavor, no further publications were observed until 2001, which signifies a time of relatively low interest. Commencing in 2002, publications were regularly released. A significant increase was observed in 2004 and 2005, during which four to five studies were published each year [12–16]. From 2006 to 2010, there was a continuous increase in MiECC research, with an average of three to six publications each year [17–36]. The years 2011 to 2019 constitute a consolidation phase, characterized by relative stability in publication numbers, typically ranging from four to seven annually. Over these years, MiECC was evaluated in diverse centers and patient populations, and there was also a rise in multicenter studies. Interest subsequently increased from 2020 onwards. The consequences of the COVID-19 pandemic (2020–2021) on the MiECC literature are especially relevant [37–81]. Although the number of publications in 2020 was relatively small, the output peaked in 2021 with seven studies, the highest in the entire period. This indicates that research on the clinical results of MiECC increased despite pandemic-related challenges. [37–66]. Therefore, the increase in publications in 2021 can be attributed both to the completion of delayed studies and to researchers prioritizing the potential benefits of MiECC under pandemic conditions. Despite a decrease to two publications in 2024, three studies were published in 2025. Please note that data from 2025 may be incomplete, given that the year is ongoing. The literature indicates a consistent increase beginning in 2002, culminating in 2021. This pattern suggests increasing acceptance of MiECC in cardiac surgical practice, prompting multicenter research (Figure 2). The first report on the geographical distribution of MiECC literature came from the Czech Republic in 1990. France took the lead in the early 2000 s, establishing a consistent publication output. In the late 2000 s, Germany and Italy made considerable contributions, thereby broadening the research network. In the 2010 s, Greece's participation further solidified MiECC research's consolidation as a Europe-focused initiative [52–81]. The surge in publications in 2021 stemmed from multiple centers concurrently contributing, thereby highlighting the increasingly multicenter characteristic of MiECC research. Concurrently, the effects of the pandemic on scientific research are apparent. The premature termination of the COMICS trial, resulting from COVID-19, provides a clear example of how the global health crisis directly impeded the generation of evidence [82]. Throughout this timeframe, trials were either halted prematurely or delayed, encompassing the MiECC field and numerous other domains of clinical research. The data presented indicate that although the pandemic induced a temporary impediment to knowledge production within cardiovascular surgery research [83–85].

### Authorship Patterns

Anastasiadis K. is a leading author in the MiECC literature, having published seven papers, the highest number among researchers. His work has emphasized systematic approaches, protocol definitions, and studies assessing the applicability of MiECC across various patient groups. Aschraf El-Essawi and Condello follow, each with three publications primarily concerning clinical outcomes and comparisons of technical variations. Furthermore, researchers including Folliguet TA, Adrian Bauer, Liu Y, Luca Koechlin, Ellam S, Ohata, Mark J. Bennett, Immer, Remadi JP, and Huybregts have each produced two publications, demonstrating the field's enduring appeal across numerous institutions. The authorship distribution in MiECC publications indicates that while several prominent individuals have shaped the literature through consistent clinical data contributions, a broad spectrum of individual investigators have also contributed. This suggests that MiECC has received attention from significant figures and independent groups across various institutions, progressively developing into a more collaborative field of scientific inquiry [10–136] .

### Relative Contribution of Countries

The analysis of country contributions to the MiECC literature reveals that Germany (20 publications), Italy (18 publications), and Switzerland (14 publications) are the top three contributors, collectively representing a significant portion of the entire body of work. Subsequently, Greece produced 11 publications and the Netherlands, 10. Significant contributions have also been made by the United Kingdom (7 publications), France (6 publications), Japan (5 publications), Finland (5 publications), and China (4 publications). Outside of the top ten countries, other nations cumulatively contributed 29 publications, representing smaller-scale input. The findings indicate that MiECC research has primarily been a European-focused area, despite increased representation from Asian nations in academic literature over time

### Evolution of Study Designs and Sample Sizes

**Fig. 3:**
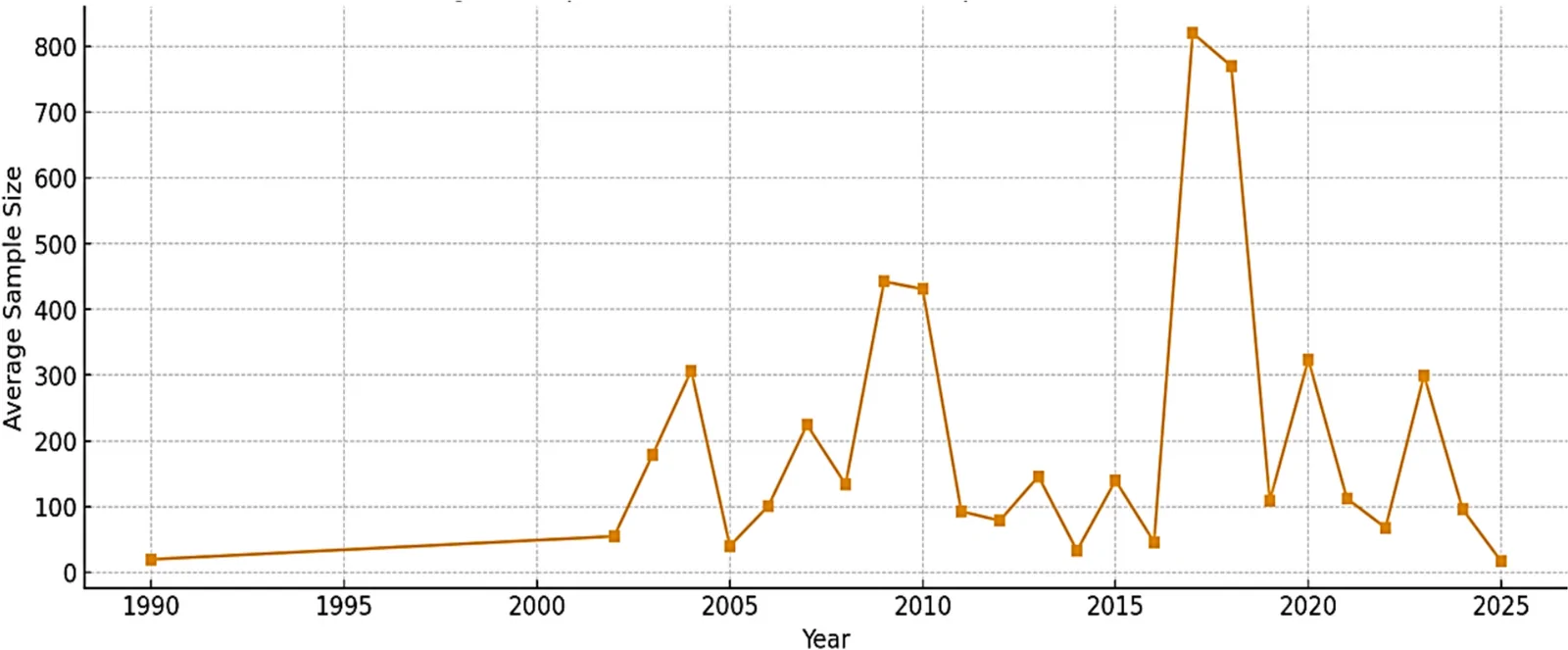
Distribution of sample sizes in MiECC studies over time

An examination of sample sizes over time reveals that patient enrollment was notably low at the beginning. The initial study, conducted in 1990, involved a limited cohort of 30 patients, and research primarily involved fewer than 50 patients until the mid-2000 s. Nonetheless, a noticeable rise in average sample sizes was seen after 2010, primarily attributable to the emergence of multicenter or prospective comparative studies. While year-to-year variability is considerable, the post-2010 period contains the largest average sample sizes recorded in the MiECC literature, with peaks exceeding 800 patients in 2017–2018, reflecting the growing contribution of multicentre and RCT. This trend indicates a shift in MiECC from pilot and exploratory applications to a method tested in larger patient populations (Figure 3).

**Fig. 4:**
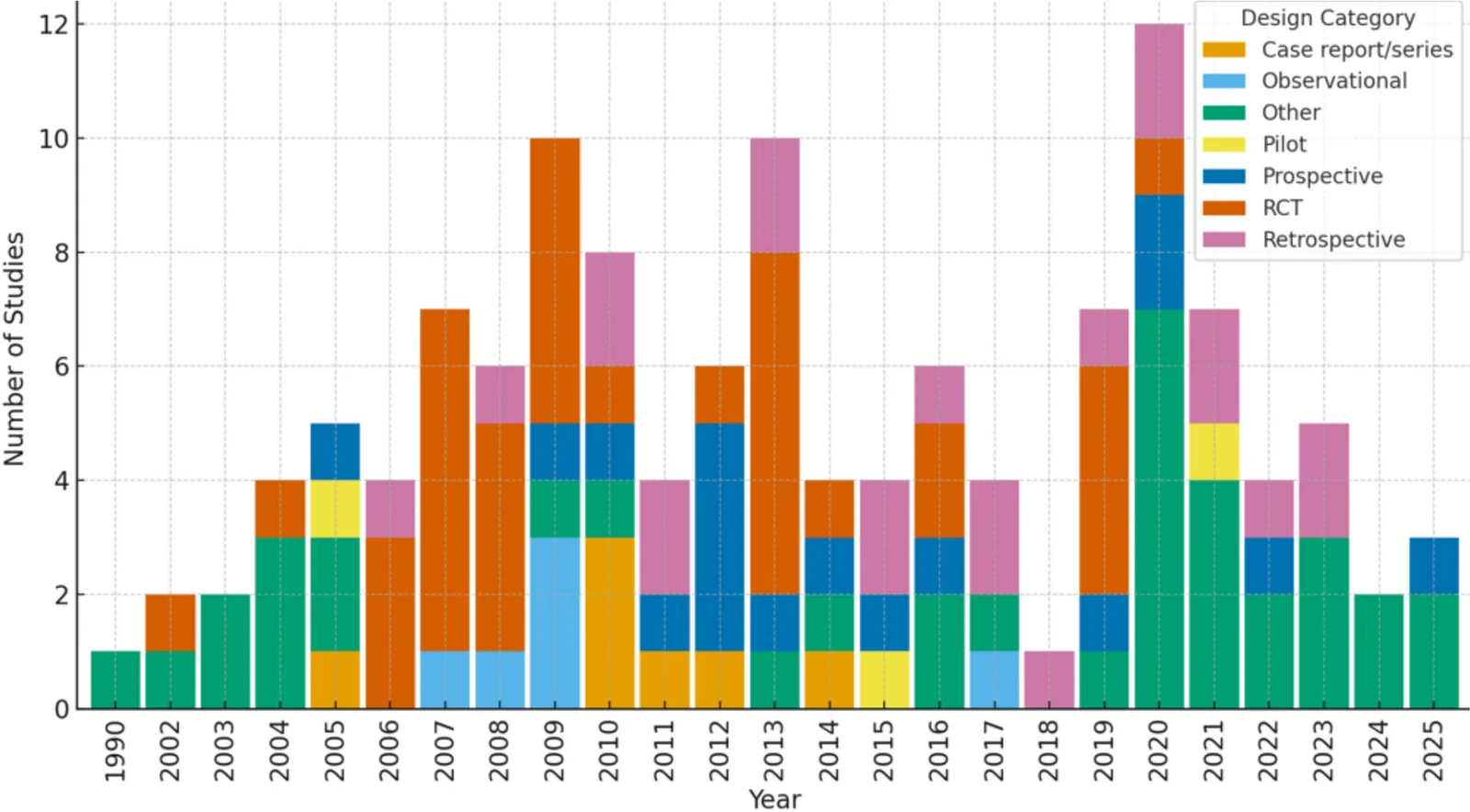
Study designs in the MiECC literature

An analysis of the study designs within the MiECC literature reveals a clear methodological evolution. The literature encompasses 36 RCT, 37 methodology/protocol papers, 22 retrospective studies, 17 prospective studies, 6 observational investigations, 7 case reports or case series, and 3 pilot studies (Figure 4). This distribution demonstrates that MiECC research progressed from small observational and case-based investigations toward larger prospective, randomised, and methodological study designs over time. The evolution of study designs reflects the maturation of the field: initial periods were characterised by case series and small observational studies, succeeded by prospective and retrospective comparative research from the mid-2000 s onwards. Beginning in the 2010 s, RCT became a regular feature in the literature, with major international multicentre trials gaining prominence in recent years. Following the early cessation of the COMICS trial due to the COVID-19 pandemic, the forthcoming MiECS RCT represents a significant opportunity to generate robust evidence regarding MiECC effectiveness and to demonstrate the resurgence of multicentre research collaboration in the post-pandemic era.

### Distribution of Surgical Procedures in MiECC Literature

**Fig. 5:**
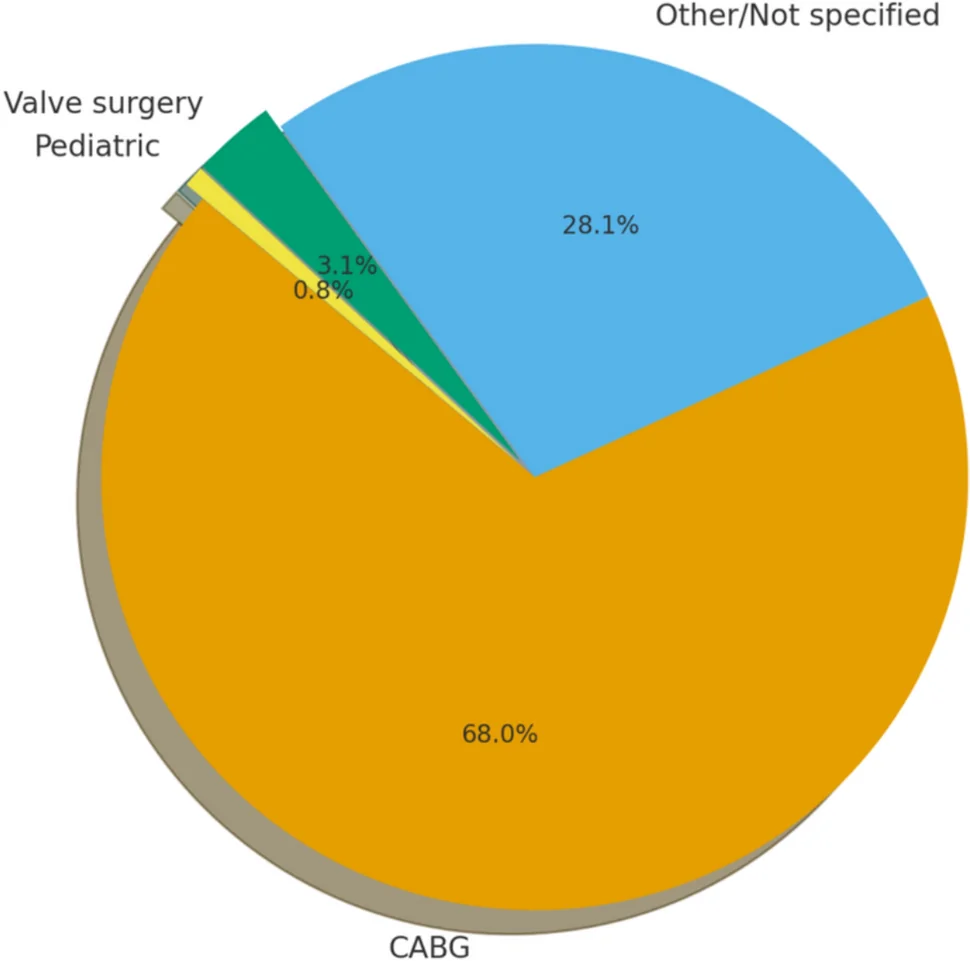
Distribution of surgical procedure

The categories presented in this section reflect the distinct surgical and physiological contexts in which MiECC has been evaluated, rather than a purely demographic classification. The paediatric category is included alongside procedure-based groups (CABG and valve surgery) because paediatric cardiac surgery involves fundamentally different circuit configurations, priming volumes, cannulation strategies, and physiological targets, rendering it a clinically distinct context of MiECC application. CABG is the most frequent surgical procedure observed in an examination of the distribution of MiECC studies. Comprising approximately two-thirds of the extant literature, this group represents the most extensively researched domain for MiECC, encompassing 87 studies. The emphasis on CABG patients is justified by the potential benefits, including decreased transfusion needs, minimized haemodilution, and a diminished inflammatory response (Figure 5). Conversely, the number of publications on valve surgery is quite limited (four studies), which may be attributed to technical variations and reduced case volumes in this particular context. Only one study has been found in the pediatric population, indicating a definite deficiency in this area. Furthermore, thirty-six studies were classified as "Other/Not Specified," mainly encompassing mixed case series or methodological/protocol reports. Consequently, this category encompasses publications founded on methodological or multicenter designs rather than a specific surgical population. The majority of MiECC research has, thus far, emphasized CABG, while data from other surgical specialities is limited. This pattern indicates that further research in valve surgery and pediatric populations, which are currently underexplored, would be significant.

### Comparison Analysis of MiECC and cCPB.

As detailed in the three-period framework described above, the comparative analysis of MiECC versus cCPB reflects this broader evolution, with study objectives and reported outcomes shifting substantially across each period. A notable recent development is the emergence of in vitro study designs within the MiECC literature. Two such studies published in 2025 (references 139–140) represent a methodological departure from purely clinical investigations, expanding the research scope toward experimental models that examine circuit design, biomaterial characteristics, and pathophysiological mechanisms. This shift is clinically relevant: as patient protection increasingly encompasses not only surgical technique but also material biocompatibility and long-term biological consequences of circuit components, laboratory-based investigation provides a complementary and necessary evidence base. It should be noted, however, that advances in surface coating technologies and biomaterials — such as heparin-bonded circuits and phosphorylcholine coatings — are not exclusive to MiECC and may equally be applied to cCPB circuits. The particular relevance to MiECC lies in its closed-circuit design, which maximises the proportional benefit of such biocompatible surfaces by eliminating residual sources of blood activation present in conventional systems.

The evolution of MiECC research can be characterised across three distinct periods. The Early Period (1990–2005) was defined by feasibility and safety investigations, comprising small case series and technical descriptions with limited patient numbers. The primary objective during this phase was to demonstrate the viability of miniaturised circuit designs and to report preliminary safety data, with outcome measures largely restricted to technical success, priming volume reduction, and basic biochemical indicators such as transfusion requirements and inflammatory markers. The Expansion Period (2006–2015) saw a marked increase in comparative clinical studies, predominantly prospective and retrospective in design, evaluating MiECC against cCPB. This period established the principal advantages of MiECC with respect to haemodilution, transfusion needs, inflammatory response, and organ function, and was characterised by growing adoption of the technique across European centres. The Maturity Period (2016–2025) is distinguished by the prominence of RCT and multicentre protocols generating higher-quality evidence. Research focus expanded beyond haematological and inflammatory endpoints to include renal protection, neurological outcomes, and long-term survival. The emergence of in vitro methodological studies in 2025 further signals a broadening of the research agenda toward circuit design, biomaterial properties, and pathophysiological mechanisms.

### Subgroup Evaluations

Subgroup analyses further assessed outcomes based on surgical procedure, high-risk patient categories, and paediatric demographics. Concerning surgical procedures, CABG was the most frequently studied. In these subjects, MiECC was significantly correlated with diminished transfusion needs, decreased haemodilution, and a less intense inflammatory reaction. In high-risk CABG scenarios, MiECC was also considered a safe option concerning hemodynamic stability. While the data on valve surgery are more restricted, the results indicate reduced haemodilution and diminished biochemical marker levels within this cohort. However, variations in cannulation methods and circuit control during valve surgery have been identified as potential determinants of the results. Among high-risk patient populations, especially the elderly, MiECC has demonstrated a reduction in perioperative complications by decreasing transfusion requirements and mitigating the inflammatory response. In individuals with kidney dysfunction, MiECC was reported to provide renal protection by minimizing postoperative creatinine increase. In patients with diminished EF (<35%), MiECC was observed to be hemodynamically well-tolerated and to provide myocardial protection, as demonstrated by reduced elevations in myocardial enzyme levels. Research on children primarily originates from prior times. The primary benefits reported within this cohort were decreased haemodilution and reduced transfusion needs, owing to lower circuit volumes. However, in light of the limited number of pediatric studies, these results necessitate validation through larger-scale investigations. Subgroup analyses indicate that the most significant advantages of MiECC have been seen in CABG patients, high-risk patients, and paediatric populations. While a clear mortality benefit has not been established, MiECC seems to offer clinical benefits concerning morbidity, biochemical markers, and the preservation of organ function (Table 2).

### Trends in Reported Primary Outcomes in MiECC Studies

**Fig. 6:**
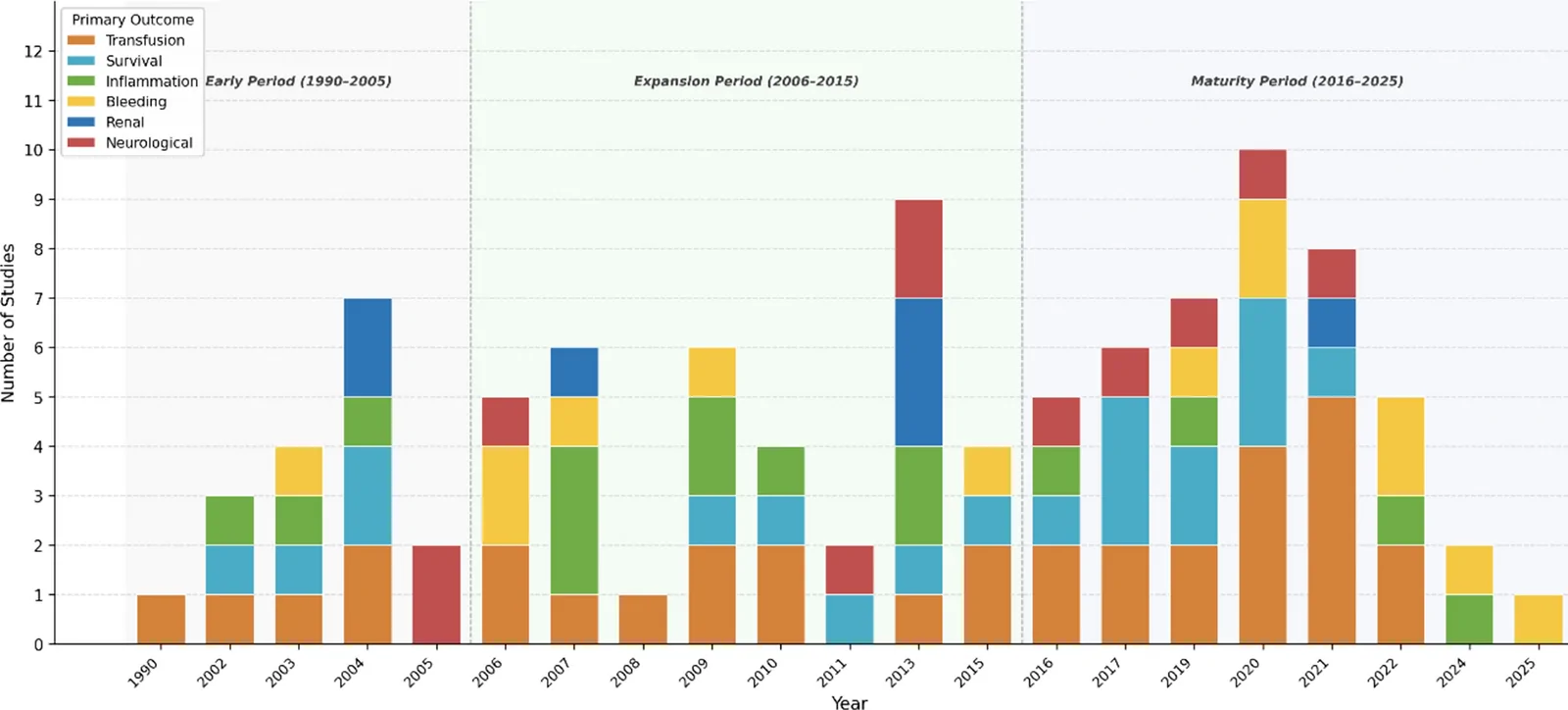
Distribution of primary outcomes reported in MiECC studies over time

The primary results reported in MiECC studies have changed over time. The parameters most often assessed are bleeding, transfusion needs, inflammatory reactions, renal and neurological complications, and survival rates. Transfusion requirements have been a key endpoint in this area since the 2000 s, with reporting frequency peaking between 2020 and 2021. Hemorrhagic outcomes were addressed less consistently but garnered attention in select studies after 2015 (Figure 6). The inflammatory response, typically assessed using biomarkers including Interleukin-6, Tumour Necrosis Factor-alpha, and C-Reactive Protein, was extensively researched beginning in the mid-2000 s, with 2013 marking the highest reporting volume. Nevertheless, a relative decrease emerged following 2020, with a shift towards outcomes of a more clinical nature. Postoperative creatinine elevation and the risk of acute kidney injury, as renal endpoints, have been systematically monitored since 2010 and were included in three studies in 2013. Neurological complications (microemboli, stroke, cognitive dysfunction) were examined across a limited number of studies, specifically in 2013, 2016, 2019, and 2020. Secondary endpoints primarily focused on survival and mortality outcomes, with a notable increase in reporting from 2017 to 2020, followed by a subsequent decline. In the initial MiECC literature, transfusion and inflammatory outcomes were prevalent, while later studies emphasized renal function and survival, although neurological outcomes received less focus. This trend indicates that MiECC research has progressively evolved from a limited focus on hematological and inflammatory advantages to expanded interests involving organ protection and long-term patient results.

## Discussion

This research systematically examined the trends, distributions of surgical procedures, primary outcomes, and subgroup analyses within MiECC literature from 1990 to 2025. The research agenda has shifted from initial safety and feasibility studies to comparative clinical trials, and, more recently, to advanced evidence from randomized controlled trials and translational research [11–136].

The data indicates that MiECC has been most extensively studied in CABG. The most consistent advantages observed in this cohort involve decreased transfusion needs, minimized haemodilution, and a reduction in the inflammatory reaction [12–66, 86–90]. Conversely, investigations into valve surgery and pediatric cohorts are limited, highlighting the requirement for additional research in these domains [32–36, 52–61, 96–105]. It is noteworthy that the "Other/Not specified" category primarily represented mixed populations or methodological/protocol studies, suggesting that MiECC research has expanded beyond clinical practice to include circuit design, biomaterials, and pathophysiological mechanisms [17–36, 67–81, 91–95, 121–130]. It should be noted that surface coating technologies including heparin-bonded circuits, phosphorylcholine coatings, and albumin-impregnated surfaces—are not exclusive to MiECC and may equally be applied to cCPB circuits. However, the clinical benefit of such biocompatible surfaces is proportionally greater in the MiECC context, as the closed-circuit architecture of MiECC eliminates the venous reservoir and cardiotomy suction—the two principal sources of residual blood activation in conventional systems. Consequently, biocompatible coatings in MiECC act synergistically with the circuit design rather than compensating for inherent architectural limitations, as is the case in cCPB. This evolving research agenda reflects growing recognition that MiECC's clinical value extends beyond perioperative haematological benefits to encompass broader organ protection and long-term patient outcomes. Recent years have witnessed meaningful advances in both circuit design and biomaterial properties within the MiECC field. Regarding surface coating technologies, heparin-bonded circuits—such as the Carmeda BioActive Surface—have been shown to reduce thrombin generation and complement activation compared with uncoated circuits, as evaluated in the 2022 double-blinded randomised controlled trial by Jenni et al. [83]. Phosphorylcholine coatings, which mimic the outer leaflet of erythrocyte membranes, represent another biocompatible surface strategy aimed at reducing platelet adhesion and inflammatory activation. Regarding pump technology, the transition from constrained vortex pumps to magnetically levitated centrifugal pumps has gained attention as a means of reducing haemolysis; this was specifically investigated in the 2021 pilot study by Condello et al., which compared magnetic levitation versus constrained vortex pump designs within a MiECC circuit [119]. Furthermore, integrated arterial filters and bubble traps within closed-circuit configurations have improved microemboli management without reintroducing blood-air contact. Most recently, the 2025 prospective pilot study by Kırali et al. [84] evaluated a hybrid MiECC system design, representing one of the first in vitro approaches to circuit optimisation within this field. Collectively, these developments indicate that MiECC is evolving beyond a purely clinical intervention toward a platform for translational research encompassing circuit engineering, biomaterial science, and pathophysiological investigation.

Analyses of subgroups demonstrated that the most significant advantages of MiECC were evident in high-risk populations, encompassing elderly patients, those with renal dysfunction, and patients with diminished ejection fraction [12–36, 86–90]. In these populations, MiECC diminished transfusion requirements, mitigated the inflammatory response, and offered both renal and myocardial protection [37–81, 91–105]. The results indicate that MiECC might be a safe alternative to cCPB in high-risk groups [52–61, 81, 91–100]. However, the evidence base pertaining to pediatric populations remains markedly restricted, thereby underscoring a crucial avenue for future inquiry [11, 32–36]. A physiologically important but underexplored aspect of MiECC's potential renal benefits concerns its interaction with goal-directed perfusion (GDP). GDP aims to tailor oxygen delivery (DO2) to the individual patient's metabolic demands during CPB, with a DO2 index threshold of approximately 280 mL/min/m^2^ widely recognised as protective against postoperative acute kidney injury [96–105]. Because MiECC is associated with significantly lower haemodilution and higher haematocrit values compared with cCPB, it inherently supports greater oxygen-carrying capacity at equivalent pump flow rates, thereby facilitating compliance with GDP thresholds. In this context, the renal protective effects observed with MiECC in the current literature may be at least partially mediated through improved oxygen delivery rather than circuit biocompatibility alone. However, it is noteworthy that the included MiECC studies rarely report DO2-related parameters systematically, representing a significant gap in the current evidence base. Recognising this gap, our group has recently conducted a study investigating the relationship between tissue oxygenation and perfusion strategy in the context of MiECC, which is currently under peer review. Future trials should incorporate GDP parameters as predefined endpoints to further clarify the mechanistic relationship between circuit design, oxygen delivery, and renal outcomes.

The COVID-19 pandemic significantly influenced MiECC research, resulting in a notable increase in multi-centre publications in 2021 [121–135]. This highlights the increasing acknowledgement of MiECC as a focal point for multicenter research. Concurrently, the premature cessation of the COMICS trial, stemming from the constraints imposed by the pandemic, accentuates the susceptibility of extensive research endeavors to worldwide emergencies [82, 85]. The upcoming MiECS trial, characterized by its substantial sample size, has the potential to generate high-quality evidence regarding the clinical efficacy of MiECC and to stimulate collaborative, multicenter research efforts in the post-pandemic era [82–85, 121–136].

A crucial element of our results pertains to how reported outcomes change over time. Initial MiECC studies mainly focused on blood and inflammatory indicators, as an attempt to prove the concept of reducing haemodilution, transfusion needs, and systemic inflammation [12–36, 86–90]. Conversely, recent research has increasingly emphasized renal, neurological, and long-term survival outcomes [37–85, 91–136]. This transition signifies the development of the evidence base, progressing from mechanistic measures to clinically relevant results. The clinical significance of this change is considerable, given that renal impairment and neurological sequelae are major indicators of adverse outcomes and overall survival after cardiac surgery. Although MiECC has not yet shown a clear mortality benefit, its multifaceted benefits, such as decreased transfusion needs, reduced inflammatory response, preserved organ function, and accelerated recovery, have increased its acceptance in clinical practice. Confirmation of these advantages in the current MiECS trial would offer robust evidence for wider application. Moreover, future investigations into pediatric surgery, valvular procedures, and neurological results are crucial for a complete assessment of MiECC's application in standard cardiac surgery.

Study Strengths and Weaknesses: This study presents several limitations. Initially, given the data's exclusive derivation from published literature, the potential for publication bias remains a concern. Additionally, in some research, there was a lack of comprehensive reporting of surgical procedures and outcome metrics, which led to a significant percentage being categorized under "Other/Not specified." Direct comparison of results is further complicated by the heterogeneity of sample sizes, patient populations, and surgical methodologies across studies. Furthermore, the majority of the included studies were single-center and medium-sized, while large multicenter randomized controlled trials are still limited. The current review did not incorporate a formal critical appraisal or risk-of-bias assessment of the included studies, as its main goal was a descriptive mapping of the MiECC literature, rather than an evaluation of intervention effectiveness. This method aligns with the scoping review methodology, which emphasizes summarizing the range, scope, and attributes of the existing evidence rather than assessing its quality. Consequently, the lack of a critical appraisal should be understood as a methodological decision consistent with the study's aims, not a deficit in methodological soundness.

## Conclusion

This scoping review provides the first comprehensive PubMed-based bibliometric and trend analysis of the MiECC literature spanning three decades (1990–2025). A total of 128 eligible studies were identified, revealing a clear and meaningful evolution in both research activity and clinical focus over time.

MiECC research originated in Europe and has remained predominantly Europe-centric, with Germany, Italy, Switzerland, Greece, and the Netherlands accounting for the largest share of publications. The field progressed through three distinct periods: an Early Period (1990–2005) characterised by feasibility and safety investigations; an Expansion Period (2006–2015) dominated by comparative clinical studies establishing the advantages of MiECC over cCPB with respect to transfusion requirements, haemodilution, and inflammatory response; and a Maturity Period (2016–2025) marked by large multicentre RCT and an expanding research agenda encompassing renal protection, neurological outcomes, long-term survival, and biomaterial properties. CABG remains the most extensively studied surgical context for MiECC, while valve surgery and paediatric populations are significantly underrepresented in the current literature. Subgroup analyses suggest that the most consistent clinical benefits of MiECC are observed in high-risk patients, including the elderly, those with renal dysfunction, and individuals with reduced EF. Although no clear mortality benefit has been demonstrated to date, MiECC offers meaningful advantages in terms of reduced transfusion requirements, attenuated systemic inflammatory response, and improved organ protection. Future research should prioritise expanding the evidence base in paediatric cardiac surgery and valvular procedures and the integration of goal-directed perfusion parameters within MiECC protocols.

## Supplementary Information

Below is the link to the electronic supplementary material.

Supplementary file1 (DOCX 90 KB)

## Data Availability

The data that support the findings of this study are available from the corresponding author upon reasonable request.
