# Supplementary material for: A Trends Analysis and Scoping Review: Minimal Invasive Extracorporeal Circulation
Source: Cardiovasc Eng Technol. 2026 Jun 25;17(5):637–53. doi: 10.1007/s13239-026-00845-x (PMC13723697; doi:10.1007/s13239-026-00845-x)
Supplement: Supplementary file 1 [file 13239_2026_845_MOESM1_ESM.docx]

**Preferred Reporting Items for Systematic reviews and Meta-Analyses extension for Scoping Reviews (PRISMA-ScR) Checklist**

| **SECTION** | **ITEM** | **PRISMA-ScR CHECKLIST ITEM** | **REPORTED ON PAGE #** |
| --- | --- | --- | --- |
| **TITLE** | | | |
| Title | 1 | Identify the report as a scoping review. | Partially. The manuscript is identified as a bibliometric trend analysis, which corresponds to a scoping review methodology, but the term “scoping review” is not explicitly stated in the title. It is clarified in the methods and abstract. |
| **ABSTRACT** | | | |
| Structured summary | 2 | Provide a structured summary that includes (as applicable): background, objectives, eligibility criteria, sources of evidence, charting methods, results, and conclusions that relate to the review questions and objectives. | Yes. The abstract is structured and includes background, objectives, eligibility criteria, sources of evidence (PubMed/MEDLINE up to September 2025), charting methods (data extraction by two independent reviewers), results (publication trends, study designs, outcomes, subgroups), and conclusions that address the review objectives. |
| **INTRODUCTION** | | | |
| Rationale | 3 | Describe the rationale for the review in the context of what is already known. Explain why the review questions/objectives lend themselves to a scoping review approach. | Yes. The rationale is described in the Introduction: although numerous MiECC studies exist, they are heterogeneous in design, patient population, and reported outcomes, and no comprehensive mapping of their temporal, geographical, and methodological trends has been conducted. These objectives lend themselves to a scoping review approach, as the aim is to provide an overview of the breadth and evolution of the evidence rather than to synthesise effect sizes. |
| Objectives | 4 | Provide an explicit statement of the questions and objectives being addressed with reference to their key elements (e.g., population or participants, concepts, and context) or other relevant key elements used to conceptualize the review questions and/or objectives. | Yes. The objectives are explicitly stated: to comprehensively analyse the temporal, geographical, methodological, and clinical trends of MiECC research. The review question was framed around the following key elements: (1) Population: human patients undergoing cardiac surgery with CPB; (2) Concept: use of MiECC systems compared with conventional CPB or alternative techniques; (3) Context: original studies published between 1990 and 2025 indexed in PubMed/MEDLINE. |
| **METHODS** | | | |
| Protocol and registration | 5 | Indicate whether a review protocol exists; state if and where it can be accessed (e.g., a Web address); and if available, provide registration information, including the registration number. | No formal review protocol was registered for this study. The methodology was predefined by the authors, but the review was not registered in PROSPERO or other databases. |
| Eligibility criteria | 6 | Specify characteristics of the sources of evidence used as eligibility criteria (e.g., years considered, language, and publication status), and provide a rationale. | Yes. Eligibility criteria specified that original human studies published in English between 1990 and September 2025 were included. Only full-text peer-reviewed articles were considered. Reviews, meta-analyses, editorials, letters, guidelines, animal studies, and abstracts without full text were excluded. The rationale was that MiECC was first introduced in the 1990s, English was selected for feasibility and consistency, and limiting to full-text peer-reviewed studies ensured data reliability and quality. |
| Information sources* | 7 | Describe all information sources in the search (e.g., databases with dates of coverage and contact with authors to identify additional sources), as well as the date the most recent search was executed. | Yes. The information source was PubMed/MEDLINE, searched from 1990 to September 2025. The most recent search was executed in September 2025. In addition, the reference lists of included studies and relevant reviews were screened manually to identify potentially eligible studies. No contact with study authors and no grey literature searches were performed. |
| Search | 8 | Present the full electronic search strategy for at least 1 database, including any limits used, such that it could be repeated. | Present the full electronic search strategy for at least 1 database, including any limits used, such that it could be repeated. |
| Selection of sources of evidence† | 9 | State the process for selecting sources of evidence (i.e., screening and eligibility) included in the scoping review. | Yes. The full electronic search strategy for PubMed/MEDLINE is presented in the Methods section, including all search terms, Boolean operators, and limits (humans, English language, exclusion of reviews, meta-analyses, editorials, letters, comments, and guidelines). The search covered studies published between 1990 and September 2025, and can be fully replicated. |
| Data charting process‡ | 10 | Describe the methods of charting data from the included sources of evidence (e.g., calibrated forms or forms that have been tested by the team before their use, and whether data charting was done independently or in duplicate) and any processes for obtaining and confirming data from investigators. | Click here to enter text. |
| Data items | 11 | List and define all variables for which data were sought and any assumptions and simplifications made. | Yes. Data were charted using a standardised Excel form created by the team. Two reviewers (DH, HK) independently extracted data, and discrepancies were resolved by a third reviewer (SOK). No contact with study investigators was undertaken. The following variables were extracted: year of publication, country, sample size, study design, type of surgical procedure, comparator groups, primary and secondary outcomes (transfusion, bleeding, inflammatory and coagulation markers, renal and neurological complications, myocardial protection, survival, ICU/hospital stay), and technological components of the MiECC system (type, coating, pump, oxygenator). When information was not clearly reported, assumptions were made (e.g., mixed or unspecified procedures were grouped as “Other/Not specified”) |
| Critical appraisal of individual sources of evidence§ | 12 | If done, provide a rationale for conducting a critical appraisal of included sources of evidence; describe the methods used and how this information was used in any data synthesis (if appropriate). | Not applicable. No formal critical appraisal of included studies was conducted, as the purpose of this review was to provide a descriptive mapping of the MiECC literature rather than to evaluate the effectiveness of interventions. This approach is consistent with the scoping review methodology, where the focus is on the breadth and characteristics of the evidence base. |
| Synthesis of results | 13 | Describe the methods of handling and summarizing the data that were charted. | Yes. Extracted data were entered into a standardised Excel sheet and classified according to year of publication, country, sample size, study design, surgical procedure, comparator group, clinical outcomes, and technological features of MiECC circuits. Data were summarised descriptively using counts and percentages. Trends were visualised with tables, heat maps, and line charts to illustrate temporal, geographical, and methodological patterns. No formal meta-analysis or inferential statistics were performed, as the aim was to provide a descriptive overview. |
| **RESULTS** | | | |
| Selection of sources of evidence | 14 | Give numbers of sources of evidence screened, assessed for eligibility, and included in the review, with reasons for exclusions at each stage, ideally using a flow diagram. | Yes. A total of 151 records were identified through PubMed. After title and abstract screening, 23 records were excluded (reviews, meta-analyses, editorials, letters, guidelines, animal studies, and conference abstracts without full text). Finally, 128 original studies were included in the analysis. The selection process is illustrated in the PRISMA-ScR flow diagram (Figure 1) |
| Characteristics of sources of evidence | 15 | For each source of evidence, present characteristics for which data were charted and provide the citations. | Yes. For each included study, the following characteristics were charted: year of publication, country, study design, sample size, type of surgical procedure, comparator group, reported clinical outcomes (e.g., transfusion, bleeding, inflammatory markers, renal and neurological complications, survival, ICU/hospital stay), and technological features of the MiECC circuits (e.g., closed-loop, low priming volume, coating, pump/oxygenator type). These data are presented in Table X (Results section), together with the full citations of all included studies. |
| Critical appraisal within sources of evidence | 16 | If done, present data on critical appraisal of included sources of evidence (see item 12). | Not applicable. No critical appraisal of included studies was performed, consistent with the scoping review methodology. Therefore, no data on quality assessment or risk of bias are presented. |
| Results of individual sources of evidence | 17 | For each included source of evidence, present the relevant data that were charted that relate to the review questions and objectives. | Yes. For each included study, the charted data presented correspond to the review objectives, namely mapping the temporal, geographical, methodological, and clinical trends of MiECC research. Specifically, data were extracted and tabulated on publication year, country of origin, study design, sample size, surgical procedure, comparator groups, reported clinical outcomes (transfusion, bleeding, inflammatory and coagulation markers, renal and neurological complications, survival, ICU/hospital stay), and technological features of MiECC circuits (closed-loop, low priming volume, coating, pump/oxygenator type). These data are presented in Results section with full citations for each study, enabling direct linkage to the review questions and objectives. |
| Synthesis of results | 18 | Summarize and/or present the charting results as they relate to the review questions and objectives. | Yes. The charting results were summarised and presented in direct relation to the review objectives. Specifically, the analysis showed (1) temporal trends, with publications increasing after 2000 and peaking in 2021; (2) geographical trends, with most contributions from European centres (Germany, Italy, Switzerland, Greece, the Netherlands); (3) methodological patterns, including 36 randomised controlled trials and 37 methodological/protocol papers, with limited multicentre studies; (4) surgical focus, predominantly on CABG, with fewer studies on valve or paediatric populations; and (5) outcome evolution, shifting from transfusion and inflammatory parameters in early studies towards renal, neurological, and survival outcomes in more recent years. Subgroup analyses suggested particular benefits in elderly patients, those with renal dysfunction, and those with low ejection fraction. These findings are presented in Tables X–Y and Figures 1–3 of the Results section, aligned with the scoping review objectives. |
| **DISCUSSION** | | | |
| Summary of evidence | 19 | Summarize the main results (including an overview of concepts, themes, and types of evidence available), link to the review questions and objectives, and consider the relevance to key groups. | Yes. The main results demonstrate that MiECC research has grown substantially since the 1990s, with 128 original studies identified, predominantly from European centres. While most studies focused on CABG, other procedures such as valve or paediatric surgery remain underrepresented. The evidence base spans feasibility series, observational cohorts, methodological/protocol reports, and 36 randomised controlled trials. The thematic focus of outcomes has shifted from transfusion and inflammatory markers in earlier years to renal, neurological, and long-term survival outcomes in more recent studies. Subgroup analyses suggest particular benefits in elderly patients, those with renal dysfunction, and those with low ejection fraction. These findings directly address the review objectives of mapping temporal, geographical, methodological, and clinical trends in MiECC research. They are highly relevant to clinicians considering the adoption of MiECC, to researchers planning future trials, and to guideline committees evaluating the integration of MiECC into routine practice. |
| Limitations | 20 | Discuss the limitations of the scoping review process. | The scoping review process itself has several limitations. Only one database (PubMed/MEDLINE) was searched, and studies published in languages other than English were excluded, which may introduce selection and language bias. Grey literature such as conference abstracts, theses, and unpublished data was not considered. No formal critical appraisal of included studies was performed, in line with the scoping review methodology, but this limits assessment of the strength or quality of the evidence. Furthermore, due to heterogeneity across study designs and outcomes, no quantitative synthesis or meta-analysis was possible. Finally, as a scoping review, the findings provide a descriptive mapping of evidence rather than definitive conclusions about clinical effectiveness. |
| Conclusions | 21 | Provide a general interpretation of the results with respect to the review questions and objectives, as well as potential implications and/or next steps. | The results show that MiECC research has expanded substantially since the 1990s, evolving from feasibility reports to multicentre randomised controlled trials. This trend analysis directly addressed the review objectives by mapping temporal, geographical, methodological, and clinical patterns. The findings suggest that while MiECC reduces transfusion requirements and inflammatory responses and may protect organ function, evidence on hard endpoints such as mortality remains limited. The implications are threefold: for clinicians, MiECC should be considered as a promising but still selectively applied alternative to conventional CPB; for researchers, further studies are required in underrepresented areas such as valve and paediatric surgery and in high-risk populations; and for policymakers, standardisation and support of large multicentre trials will be key to integrating MiECC into future guidelines. Next steps should therefore focus on long-term outcomes, broader surgical applications, and technological refinement of MiECC systems. |
| **FUNDING** | | | |
| Funding | 22 | Describe sources of funding for the included sources of evidence, as well as sources of funding for the scoping review. Describe the role of the funders of the scoping review. | Funding information for the included studies was not systematically extracted, as the focus of this review was on bibliometric and trend analyses. This scoping review received no specific funding. The authors conducted the study independently, and no external funder had any role in study design, data collection, analysis, interpretation, or the decision to publish. |

JBI = Joanna Briggs Institute; PRISMA-ScR = Preferred Reporting Items for Systematic reviews and Meta-Analyses extension for Scoping Reviews.

* Where *sources of evidence* (see second footnote) are compiled from, such as bibliographic databases, social media platforms, and Web sites.

† A more inclusive/heterogeneous term used to account for the different types of evidence or data sources (e.g., quantitative and/or qualitative research, expert opinion, and policy documents) that may be eligible in a scoping review as opposed to only studies. This is not to be confused with *information sources* (see first footnote).

‡ The frameworks by Arksey and O’Malley (6) and Levac and colleagues (7) and the JBI guidance (4, 5) refer to the process of data extraction in a scoping review as data charting*.*

§ The process of systematically examining research evidence to assess its validity, results, and relevance before using it to inform a decision. This term is used for items 12 and 19 instead of "risk of bias" (which is more applicable to systematic reviews of interventions) to include and acknowledge the various sources of evidence that may be used in a scoping review (e.g., quantitative and/or qualitative research, expert opinion, and policy document).

*From:* Tricco AC, Lillie E, Zarin W, O'Brien KK, Colquhoun H, Levac D, et al. PRISMA Extension for Scoping Reviews (PRISMAScR): Checklist and Explanation. Ann Intern Med. 2018;169:467–473. [doi: 10.7326/M18-0850](http://annals.org/aim/fullarticle/2700389/prisma-extension-scoping-reviews-prisma-scr-checklist-explanation).
